# Facile Fabrication of Nanocellulose Beads with Tunable Carboxyl Content for Blood Purification

**DOI:** 10.3390/polym18131647

**Published:** 2026-07-02

**Authors:** Zhongqiu Ge, Hengfeng Zhu, Yiyang Chen, Yihang Rong, Zhuqun Shi, Quanling Yang

**Affiliations:** 1 School of Chemistry, Chemical Engineering and Life Sciences, Wuhan University of Technology, Luoshi Road 122, Wuhan 430070, China; gzq320921@163.com (Z.G.); c1371858398@163.com (Y.C.); 352909@whut.edu.cn (Y.R.); 2 School of Materials Science and Engineering, Wuhan University of Technology, Luoshi Road 122, Wuhan 430070, China; 15972061174@163.com

**Keywords:** nanocellulose aerogel, adsorbent, blood purification, bilirubin

## Abstract

Most adsorbent materials typically face difficulties such as poor blood compatibility, weak mechanical strength, and high cost. In this study, oxidized 2,2,6,6-tetramethylpiperidine-1-oxyl (TEMPO) was used to obtain cellulose nanofiber (TOCN), and cellulose beads were prepared using a drop curing method. The structure, adsorption properties, and blood compatibility of the prepared beads were thoroughly investigated. The TOCN beads exhibit a uniform, nanometer-scale, three-dimensional porous structure. With increasing carboxyl content, after adsorption of TOCN beads, the bilirubin concentration in rabbit plasma decreased from 0.03 to 0.0089 mg mL^−1^ within 90 min, which is significantly lower than the average bilirubin concentration in humans (about 0.01 mg mL^−1^), and the bilirubin concentration decreased by about 70%. The results illustrated the excellent blood compatibility, self-anticoagulant ability, and superior toxin removal capabilities of the TOCN beads, highlighting their potential as an ideal blood purification adsorbent.

## 1. Introduction

Chronic kidney disease and liver disease have compromised the health of many people, and patients with end-stage renal disease and cardiovascular disease are vulnerable to death. Due to the high cost and the lack of kidney donors, kidney transplantation can only support a small number of people [1,2,3]. Blood purification technology has become the main treatment technology for patients with chronic kidney disease [4,5]. When the kidneys fail, toxins build up in the body, leading to increased mortality and morbidity. Small water-soluble molecules can be removed by dialysis, but medium-sized molecular toxins are difficult to remove. According to differences in principles, blood purification is generally divided into hemodialysis (HD) [6], hemofiltration (HF) [7], and hemoperfusion (HP) [8]. Among them, HD therapy is more effective in removing toxins with small and large molecular weights, and this is an area of wide concern. Traditional adsorbent materials, such as activated carbon and graphene, have poor mechanical strength and poor blood compatibility, which will cause side effects in the human body; thus, these materials do not meet the requirements of blood purification adsorbent [9,10]. Biomass materials are widely studied in the field of blood purification because of their biocompatibility, which traditional polymer materials do not have [11]. Among them, chitosan has good biocompatibility, hydrophilicity, and degradability [12]. Agarose–chitosan cryogenic gel with glutarglycol as crosslinking agent was studied for the purification of immunoglobulin (IgG). The results showed that the maximum adsorption capacity of the frozen gel for IgG was 71.4 mg/g, and it showed feasible stability [13]. However, formaldehyde and pentanediol, as crosslinking agents, are harmful in aqueous solutions [14]. Amino-functionalized polyethylene-co-vinyl (PVA-co-PE) nanofiber membranes were developed to adsorb bilirubin from solutions containing bovine serum albumin (BSA) [15]. Its adsorption capacity for bilirubin in 40 g/L BSA solution was 35 mg/g. However, some human proteins could also be adsorbed to PVA-co-PE nanofiber membranes through electrostatic interactions, resulting in low adsorption of bilirubin. Cellulose nitrate (CN) with a low nitrification degree was synthesized using the denitrification reaction. Secondary amine cellulose nitrate (CAN) and quaternary amine cellulose nitrate (CCN) derivatives were synthesized using CN as the substrate and used as the adsorbents for creatinine. The adsorption capacities were 2.04, 1.47 and 1.08 mg/g, separately. The kind of adsorbents are not affected by urea content, but their adsorption effect is not very ideal [16]. On the one hand, blood purification adsorbent materials need excellent blood compatibility to reduce the side effects in the human body. On the other hand, a higher adsorption capacity is required to increase the removal effect of toxins. Since blood purification technology has been proposed, people have been focusing on adsorption properties and blood compatibility to study new blood purification adsorbents.

Among polysaccharides, cellulose is the most abundant on earth and can be obtained from various plant substrates [17]. The natural polymer cellulose is inexpensive, non-toxic, biodegradable, renewable, and biocompatible [18]. Cellulose molecules have many hydroxyl groups and are highly hydrophilic. The size of nanocellulose material in one dimension is 1–100 nm [19]. Nanocellulose (TOCN) material with a high specific surface area and a large aspect ratio was obtained using the TEMPO oxidation process [20]. The oxidation reaction changes the alcohol hydroxyl group on the surface of cellulose to form the carboxyl group, thus showing higher electricality. Due to electrostatic repulsion, TOCN can form a staggered three-dimensional network structure, which increases its specific surface area [21]. Therefore, TOCN can be used as a potential adsorbent material for blood purification.

In this work, the TEMPO oxidation method was used, and the amount of NaClO was adjusted to obtain TOCN with different carboxyl concentrations. Nanocellulose beads (TOCNB) were obtained using the method of drip curing under the action of CaCl_2_ crosslinking. The microstructure and chemical structure of TOCN beads were studied. Using bilirubin (BR), creatinine, uric acid, and Cu^2+^ as models, the adsorption properties of different carboxyl groups and specific surface areas were studied. Based on the adsorption model, the adsorption mechanism of blood toxin was analyzed to explore its blood compatibility, and the feasibility of nanocellulose beads as adsorbents for blood purification was discussed.

## 2. Materials and Methods

### 2.1. Materials and Chemicals

Softwood bleached kraft pulp (SBKP) was provided by Nippon Paper Industries (Tokyo, Japan). NaClO, creatinine, and uric acid were obtained from Aladdin (Shanghai, China). 2,2,6,6-tetramethyl-1-oxyl (TEMPO) was supplied from Sigma-Aldrich Corporation (St. Louis, MO, USA). NaBr, NaOH, bilirubin (BR), copper sulfate, and hydrochloric acid were of analytical grade and obtained from Sinopharm Chemical Reagent Co., Ltd. (Shanghai, China). PBS was bought from Thermerfeld Biochemical Products Co., Ltd. (Thermerfeld, Germany).

### 2.2. Preparation of TOCN Beads

**Figure 1:**
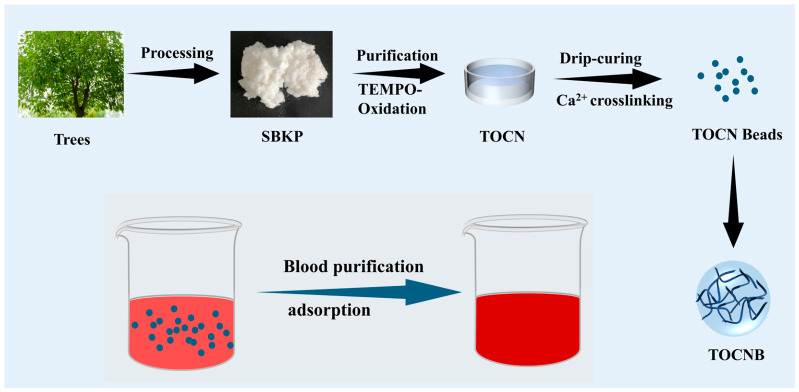
Fabrication process of TOCN beads.

TOCN beads with carboxylate groups were prepared from SBKP using the TEMPO oxidation method. As illustrated in Figure 1, 0.2 g NaBr, 0.032 g TEMPO, and 2 g SBKP were added to 200 mL of deionized water and stirred. In order to obtain TOCN with different carboxyl contents, the amount of sodium hypochlorite was changed (5, 7.5, and 10 mmol/g cellulose), and the pH of the system was maintained at about 10 for 3 h by adding 0.5 M NaOH. The obtained TEMPO-oxidized pulp was washed with deionized water and dispersed with a high-pressure homogenizer (microchannel, 30 MPa) to obtain a TOCN dispersion solution with a concentration of 0.55 wt % [22].

Here, 100 g nanocellulose dispersion was stirred for 30 min on a magnetic stirrer and then was evacuated for 30 min to remove the bubbles. Then, 1000 mL of 0.1 mol L^−1^ CaCl_2_ solution was prepared, and TOCN was dropped vertically into CaCl_2_ solution at uniform speed with a 1 mL syringe to obtain TOCN hydrogel beads. Twenty-four hours later, it was washed with deionized water many times, which was replaced by an ethanol gradient. Then, it was treated with 100% tert-butanol and freeze-dried. Nanocellulose hydrogel beads prepared from dispersions with different carboxylate contents were named TOCNB1, TOCNB2, and TOCNB3, separately.

### 2.3. Characterization

The morphologies and structures of the TOCN beads were obtained using a scanning electron microscope (SEM) (Hitachi S-4800, Hitachi High-Technologies Corporation, Tokyo, Japan) at 10 kV. Carboxyl group contents were determined using conductometric titration (Raycom DDS-11A, Shanghai INESA Scientific Instrument Co., Ltd., Shanghai, China) with NaOH titration of TOCN dispersions. The particle size distribution of TOCN beads was detected using a laser particle size analyzer (Malvern, Mastersizer 2000, Malvern Instruments Ltd., Malvern, UK) in an aqueous environment. The internal pore size distribution and specific surface area of TOCN beads were analyzed using a TriStar II 3020 automatic specific surface area and porosity analyzer (Micromeritics, Atlanta, GA, USA). Fourier-transform infrared (FTIR) spectroscopy was performed using a FTIR spectrometer (Thermo Fisher Scientific Nicolet 6700, Waltham, MA, USA). A zeta potentiometer (Zetasizer Nano ZS90, Malvern Instruments Ltd., Malvern, UK) was used to test the surface potential of TOCN beads in the water phase. The absorbance of TOCN beads in the wavelength range of 300 to 800 nm was recorded using an ultraviolet-visible spectrophotometer (T-UV 1810, Shanghai Yoke Instrument Co., Ltd., Shanghai, China), and the linear relation between absorbance and concentration was obtained.

All quantitative data reported herein were generated from three or more independent technical replicates per synthesized bead batch to ensure experimental robustness. Data is presented as mean ± SD.

### 2.4. Adsorption Experiments

#### 2.4.1. Adsorption Kinetics

The TOCN beads (5 mg) were dipped into a bilirubin aqueous solution (0.15 mg/mL, 10 mL) to investigate the adsorption kinetics. Then, the sample was stirred under darkness conditions at room temperature. The concentration of bilirubin solution after adsorption at different time points was measured using an ultraviolet–visible spectrophotometer (T-UV 1810, Shanghai) at 438 nm. The adsorption capacity (Q_e_) was determined using Equation (1) as follows:

(1) $$q_{e}=\frac{\left(C_{0}-C_{t}\right)V}{m}$$

where *q_e_* (mg g^−1^) is the equilibrium adsorption capacity of the adsorbent, *C*_0_ (mg mL^−1^) is the initial concentration of the adsorbed substance, *C_t_* (mg mL^−1^) is the concentration of the adsorbed substance at time t, *V* (mL) is the volume of the adsorbed substance solution, and *m* (mg) is the weight of the adsorbent.

#### 2.4.2. Adsorption Isotherms

The adsorption isotherms of the samples were acquired by equilibrating 5 mg sample in 10 mL bilirubin solution for 2 h with different initial concentrations of 0.02, 0.04, 0.06, 0.08, 0.1, 0.15 and 0.2 mg/mL. The adsorption isotherm process was similar to that for adsorption kinetics described above.

#### 2.4.3. Other Toxin Adsorption Experiments

Briefly, 5 mg TOCN aerogel beads was weighed and immersed in 10 mL 0.1 mg mL^−1^ creatinine and 10 mL 0.15 mg mL^−1^ uric acid solution, separately, and vibrated at room temperature for 12 h. After the adsorption process, creatinine and uric acid concentrations were measured at 232 nm and 293 nm. The adsorption capacity was calculated separately. Similarly, 5 mg TOCN aerogel beads was added to 10 mL 0.1 mg ml^−1^ Cu^2+^ solution. After 12 h, the concentration of Cu^2+^ was measured, and the adsorption capacity was calculated.

### 2.5. Blood Compatibility Test

The hemolysis rate of TOCN beads with different carboxyl groups was determined using a UV–Vis spectrophotometer. The TOCN beads were cleaned with PBS and saline. A specific amount of anticoagulant whole blood was collected from rabbit anticoagulated whole blood approved by the Ethics Committee of Wuhan University of Technology (WHUT(伦)2025-079, 22 December 2025). Blood was obtained via central ear artery puncture under sterile conditions. Blood samples were centrifuged and washed at 5000 rpm for 5 min five times to obtain the red blood cells. Then, 1700 μL PBS solution was obtained and mixed well with 0.5 mL of red blood cells obtained in the preceding steps. Next, 0.5 mL diluted red blood cells were added to 2 mL PBS solution and 2 mL deionized water, which were used as a negative control and positive control, respectively. Then, 5 mg TOCN beads were immersed in 2 mL of red cell bloods in PBS solution and then incubated for 120 min at 37 °C. After the mixture was centrifuged for 3 min at 5000 rpm, the solution was visualized using a digital camera. The absorbance of the supernatant at 542 nm was measured using centrifugation at 5000 rpm for 3 min. Test each sample three times to reduce the error. The hemolysis rate was calculated according to Equation (2) as follows:

(2) $$HR=\frac{{\mathrm{OD}}_{t}-{\mathrm{OD}}_{\mathrm{nc}}}{{\mathrm{OD}}_{\mathrm{pc}}-{\mathrm{OD}}_{\mathrm{nc}}}$$

where the *HR* is the hemolysis rate of the material, and *OD_n_*, *OD_pc_*, and *OD_nc_* are the absorbance of the aerogel beads, the positive control, and the negative control, respectively.

The blood calcium time of TOCN beads was investigated using a timer. The TOCNB beads (5 mg) were incubated in platelet-poor plasma obtained by centrifuging anticoagulant whole blood at 3000 rpm for 15 min. The beads were shaken at 37 °C for 1 h. Then, 200 μL of preheated 25 mmol/L CaCl_2_ was added, and the timer was started. When white turbid fibrin appeared or plasma coagulation was observed, the timing was stopped. The time used was the calcium rehydration time of TOCN beads. All the tests were repeated three times.

## 3. Results and Discussion

### 3.1. Structural Morphology and Surface Analysis of TOCN Beads

**Figure 2:**
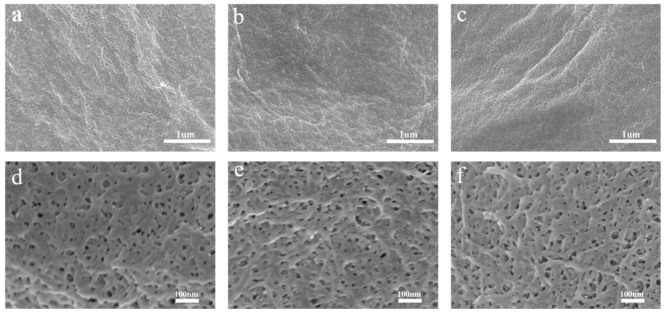
SEM images of TOCNB beads with different carboxyl contents. (**a**,**d**) TOCNB1, (**b**,**e**) TOCNB2, and (**c**,**f**) TOCNB3.

The microstructure of TOCN beads with different carboxyl contents was analyzed using SEM. Figure 2 shows the surface morphology of TOCN beads with different carboxyl contents. Figure 2a–c present the SEM images of TOCN beads at 20,000 magnification. The TOCN beads obtained using CaCl_2_ solution as the crosslinking agent and the simple and rapid drip curing method present a very smooth surface and a porous three-dimensional network structure. TOCN can form gels with a crosslinker rapidly and has a large contact area. The gel formation rate of TOCN was faster with the increase in carboxyl content. Figure 2d–f shows an enlarged SEM image of TOCN beads. Due to the interconnection between TOCN, a uniform, nanoscale three-dimensional porous structure was observed on the surface of TOCN beads. In addition, the pore size of TOCNB beads ranges from a few nanometers to tens of nanometers and belongs to a mesoporous structure. This is conducive to the adsorption of TOCN beads on toxic molecules.

### 3.2. The Carboxy Group Content of TOCN

**Figure 3:**
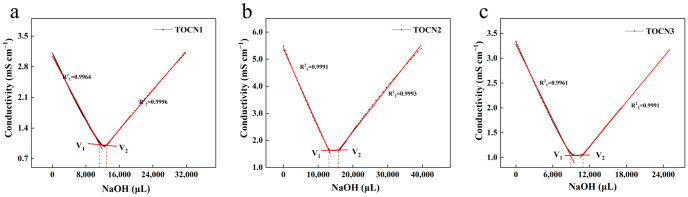
The carboxy group content of (**a**) TOCN1, (**b**) TOCN2, and (**c**) TOCN3.

To verify the effect of carboxyl group content on the adsorption performance of TOCN beads, the carboxyl group contents of three TOCN samples with different oxidation degrees were determined using conductometric titration. The titration curves are shown in Figure 3, which exhibited a characteristic V-shaped profile. The intersection points of the linear segments indicate the equivalence points, corresponding to the complete neutralization of carboxyl groups. The carboxyl group contents of TOCN1, TOCN2, and TOCN3 were 1.3 mmol/g, 1.5 mmol/g, and 1.7 mmol/g, respectively. The carboxyl group contents increased with the increasing NaClO dosage, which is consistent with the previous literature [19].

### 3.3. Specific Surface Area and Pore Size Distribution of TOCN Beads

**Figure 4:**
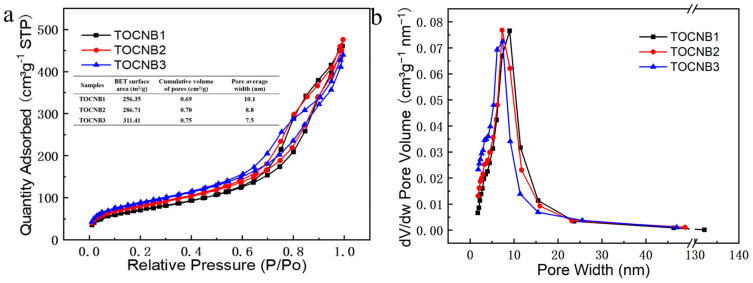
(**a**) N_2_ adsorption–desorption isotherm of TOCN beads. (**b**) Pore size distribution curves.

The specific surface area and pore size distribution of adsorbents are important parameters for blood purification. The nitrogen isothermal adsorption and desorption curves of TOCN beads are shown in Figure 4a. It is worth noting that pores can be generally classified into mesoporous (2–50 nm) and microporous (less than 2 nm) sizes. The nitrogen adsorption capacity of TOCN beads increased with the increasing relative pressure. The adsorption of nitrogen by TOCN beads was multi-layer adsorption. These results indicate that mesoporous or microporous TOCN beads were dominant, and the carboxyl content of different TOCN beads were different. According to the BET calculation model, the specific surface areas of TOCNB1, TOCNB2, and TOCNB3 gradually increased from 256.35 m^2^/g to 311.41 m^2^/g. Due to the increase in carboxyl content in nanocellulose, the specific surface area of TOCN beads increased, which made a significant contribution to the structure of the beads. The pore size distribution curve of adsorbent can be obtained using the Barrett–Joyner–Halenda (BJH) model [23]. Figure 4b shows the pore size distribution curve of TOCNB. From the analysis of pore size distribution, the average pore sizes of TOCNB1, TOCNB2, and TOCNB3 were 10.1 nm, 8.8 nm, and 7.5 nm, respectively. Its pore size is mainly distributed in the range of 5~15 nm and is categorized as a general mesoporous material structure, which verifies the SEM imaging results. The results showed that with the increase in carboxyl group content, TOCN beads formed more pores, larger specific surface areas, and a three-dimensional porous network structure. This makes the specific surface area of TOCN beads adjustable, which has certain application prospects in the field of blood purification adsorbents [24].

### 3.4. Chemical Structure Analysis of TOCN Beads

**Figure 5:**
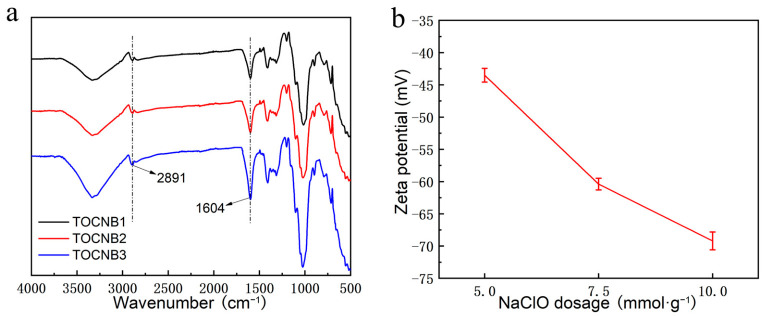
(**a**) FT-IR spectra of TOCN beads with different carboxyl contents. (**b**) Zeta potential values of different TOCN beads.

Figure 5a shows the FTIR spectra of TOCN beads with different carboxyl contents. It can be seen that the characteristic absorption peak at 3336 cm^−1^ of the sample corresponds to the stretching vibration of -OH in TOCN. The peak at 2891 cm^−1^ was ascribed to the C-H stretching on TOCN. The peaks at 1604 cm^−1^ and 1024 cm^−1^ represent the asymmetric stretching of -COO^−^ and stretching of C-O, respectively [25]. In the spectrum of TOCN beads, the peak of the asymmetric stretching of -COO^−^ and the stretching vibration of O-H moved toward the high wave number. With the increase in NaClO content in the preparation process, the oxidation degree of the hydroxyl group in TOCN was deeper, and the content of the carboxyl group was increasing. In addition, with the increase in the carboxyl group content the hydrogen bond interaction force between TOCN molecules weakened, resulting in a larger O-H stretching vibration frequency of TOCN. The zeta potentials of different TOCN samples was measured using a zeta potentiometer. The zeta potential values of TOCN1, TOCN2, and TOCN3 are shown in Figure 5b. The potential values were −43.5mV, −60.4mV, and −69.2mV, respectively. This shows that as the content of carboxyl group increases, the negative charge of TOCN increases. The results proved the successful preparation of TOCN beads with different carboxyl contents.

### 3.5. Adsorption Kinetics of TOCN Beads

**Figure 6:**
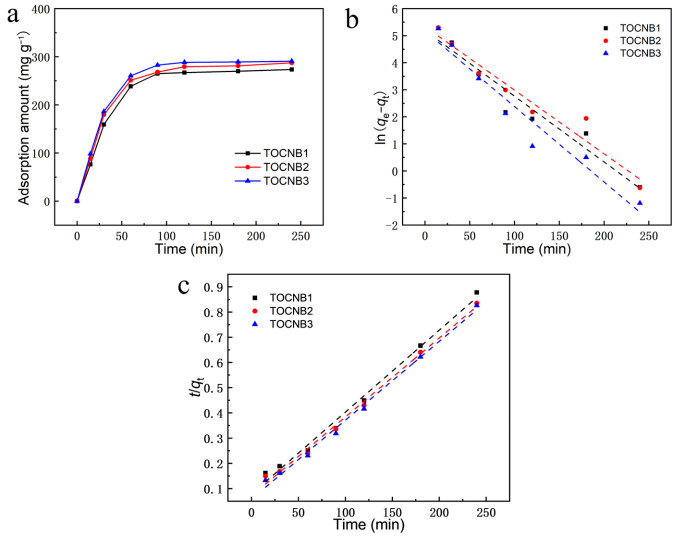
(**a**) Adsorption kinetics curves of TOCN beads for bilirubin adsorption. (**b**) The linear plot of the pseudo-first-order kinetic model and (**c**) linear plot of pseudo-second-order kinetic model of TOCN beads.

Using bilirubin as the main model, the adsorption effect of adsorbent on bilirubin was investigated. The adsorption capacity of bilirubin was the most important performance factor. As shown in Figure 6a, TOCN beads exhibited initial rapid adsorption in the first 2 h, followed by slow adsorption until equilibrium. The main reason was that at the beginning of adsorption of bilirubin molecules by adsorbent, there are more adsorption sites. The adsorption of bilirubin by adsorbent was strong. When the adsorption sites were saturated, the adsorption equilibrium of bilirubin molecules was reached. It can be seen that with the increase in carboxyl content, the adsorption rate of bilirubin by TOCN beads gradually increased, indicating that the mutual binding sites of TOCN beads increased with the increase in specific surface area and carboxyl content, leading to a longer adsorption equilibrium time. It also showed that the specific surface area and carboxyl content of TOCN beads affected the adsorption capacity of the adsorbent. In order to further study the adsorption process and mechanism of bilirubin by TOCN beads, a pseudo-first-order kinetic model and pseudo-second-order kinetic model were used to fit the adsorption data of TOCNB with time. The pseudo-first-order kinetic model is expressed as follows: *ln*(*q_e_* − *q_t_*) = *lnq_e_* − *k*_1_*t*. The pseudo-second-order kinetic model is expressed as follows: *t*/*q_t_* = 1/*k*_2_*q*^2^*_e_* + *t*/*q_e_*. Figure 6b,c shows the fitting curves, and Table 1 shows the parameters of the pseudo-first-order and second-order kinetic models. The *R*^2^ of curves the pseudo-first-order kinetic models of TOCNB1, TOCNB2, and TOCNB3 were 0.94228, 0.95002, and 0.9479, respectively. In the pseudo-second-order kinetic models of TOCNB1, TOCNB2, and TOCNB3, *R*^2^ values were 0.99171, 0.99454, and 0.99459, respectively. In addition, in the pseudo-second-order kinetic model, the saturated adsorption capacities (*q_max_*) of TOCNB1, TOCNB2, and TOCNB3 were similar to the experimental value. The results show that the adsorption process of bilirubin includes a physical process and chemisorption, and chemisorption plays a dominant role in the adsorption process [26].

### 3.6. Adsorption Isotherms of TOCN Beads

**Figure 7:**
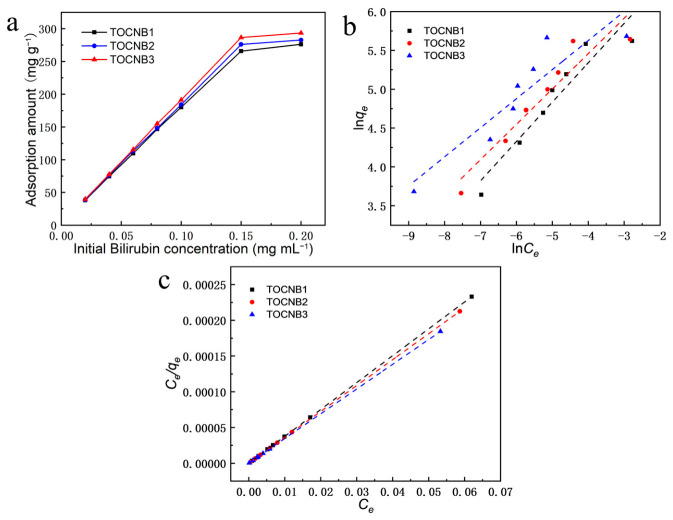
(**a**) Adsorption isotherms curves of TOCN beads for bilirubin adsorption. (**b**) Freundlich isotherms and (**c**) Langmuir isotherms of TOCN beads.

In order to further evaluate the adsorption capacity of TOCN beads on bilirubin, the adsorption isotherm model was used for fitting. As shown in Figure 7a, the adsorption capacity of TOCN beads increased rapidly with the increase in bilirubin concentration. The result was related to the mesoporous structure and the carboxyl and hydroxyl groups on the surface of TOCN beads. Subsequently, a Freundlich isotherm model (*lnq*_e_ = *lnK_F_* + *b_F_lnC_e_*) and Langmuir isotherm model (*C_e_*/*q_e_* = *C_e_*/*q_m_* + 1/*q_m_*_KL_) were used to investigate the adsorption behavior of TOCNB beads. Figure 7b,c shows the fitting curves of TOCN beads, and the adsorption isotherm parameters are shown in Table 2. The Langmuir isotherm model had the highest correlation coefficients of 0.99194, 0.99536 and 0.99571, which showed that TOCN was more in line with this model. The results also showed that the adsorption behavior of TOCN beads was a monolayer, indicating that chemisorption was based on monolayer adsorption. The -COOH and -OH groups were involved in bilirubin adsorption or hydrogen bond formation. In addition, the *b_F_* values of the prepared TOCN beads were all less than 1, indicating that TOCN beads were favorable to the adsorption process of bilirubin [27].

### 3.7. Adsorption Capacity of TOCN Beads to Other Toxins

**Figure 8:**
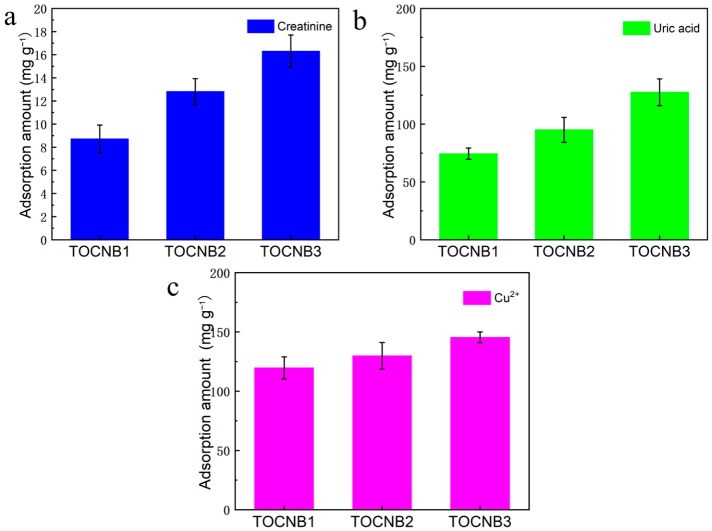
(**a**) Creatinine, (**b**) uric acid, and (**c**) Cu^2+^ adsorption amounts of TOCN beads.

Blood purification adsorbents are required to effectively remove a broad spectrum of toxins with distinct physicochemical properties. To assess the toxin removal capacity of TOCN beads, common toxins such as creatinine, uric acid, and Cu^2+^ were selected for evaluation. Figure 8a–c shows the adsorption of creatinine, uric acid, and Cu^2+^ by TOCN beads. It can be seen that the adsorption capacity of TOCNB1, TOCNB2, and TOCNB3 for creatinine increased from 8.7 mg g^−1^ to 16.3 mg g^−1^. The adsorption capacities of TOCN beads for Cu^2+^ increased from 119.7 mg g^−1^ to 145.4 mg g^−1^. The adsorption capacity of TOCN beads on cationic toxins like creatinine and Cu^2+^ is attributed to the increase in the carboxyl group content, and the toxin molecules can be adsorbed through charge interactions mainly because the -COOH and -OH on the surface of the beads provided more binding sites for the beads. In addition, the mesoporous structure of TOCN beads promoted efficient mass transfer of toxin molecules. For uric acid, the adsorption capacity of TOCN beads were 74.41 mg g^−1^, 95.08 mg g^−1^, and 127.51 mg g^−1^, respectively. Notably, TOCNB3 demonstrated the highest adsorption capacity, underscoring that an increase in carboxyl group content enhances the toxin removal ability of beads. These results indicated that TOCN beads possess broad-spectrum toxin removal capability and hold significant promise as high-performance adsorbents for blood purification applications.

### 3.8. Blood Compatibility of TOCN Beads

**Figure 9:**
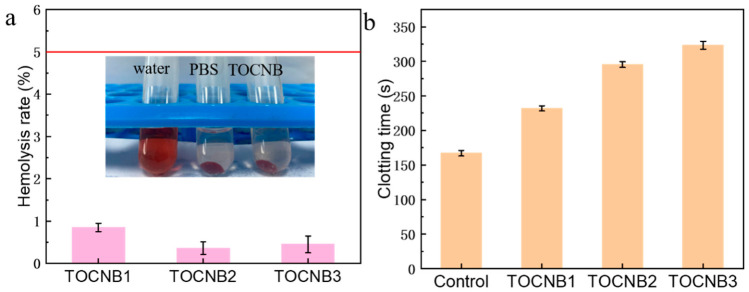
(**a**) Hemolysis rate and (**b**) clotting time of TOCN beads.

The hemolysis rate and anticoagulant properties of biomaterials are crucial parameters to evaluate blood compatibility. When biological materials come into contact with blood, due to the characteristics of their structures, forms, and composition, such materials may cause serious pollution to the blood, destroy the red blood cells in the blood, release numerous pro-coagulant factors and subsequently aggravate the clotting process, and inhibit the coagulation, adhesion, and deformation of platelets, eventually leading to thrombus generation. The blood compatibility of biomaterial can be indicated by whether it causes rupture and coagulation reactions of red blood cells. The absorbance value of biomaterials after contact with red blood cells is measured using a UV–visible spectrophotometer. Generally qualified biomaterials have a hemolysis rate (HR) of less than 5%. Figure 9a shows the hemolysis rate of TOCN beads, and the hemolysis rate (HR) of TOCNB1, TOCNB2, and TOCNB3 beads are 0.85%, 0.36% and 0.45%, respectively. This indicates that TOCN beads have a low hemolysis rate (less than 1%) and exhibit good biocompatibility. At the same time, an image of the phenomenon after hemolysis of the sample is shown in the photo in Figure 9a. Compared with the positive control, the supernatant of red blood cells after TOCN bead treatment and centrifugation was transparent, indicating that the amount of hemoglobin released by red blood cells during incubation was negligible. Its safety and non-toxicity were demonstrated. The recalcification time of TOCN beads is shown in Figure 9b. The coagulation time can be characterized by the plasma recalcification time. The more anticoagulant substances present, the longer the plasma recalcification time, and the more significant the anticoagulant property of the material. The data showed that the plasma recalcification time of TOCNB3 was nearly twice that of the blank control group. Furthermore, the plasma recalcification time of TOCN beads was prolonged with the increase in carboxyl group content. The results showed that TOCN beads had excellent anticoagulant properties and excellent blood compatibility, which makes the material have great application potential in the field of blood purification.

### 3.9. Blood Perfusion Capability of TOCN Beads

**Figure 10:**
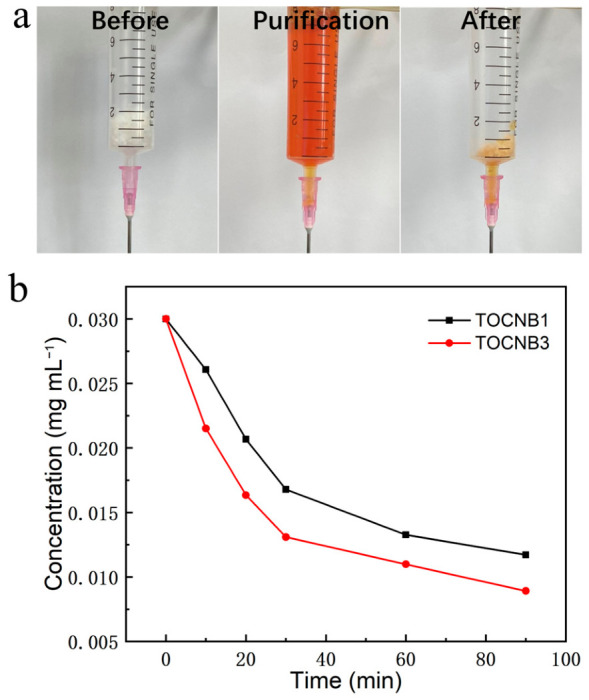
(**a**) Digital photographs of simulated hemoperfusion and (**b**) curves of bilirubin concentration.

The adsorption experiments indicate that the nanocellulose aerogel beads exhibit significant bilirubin adsorption capacity. Nanocellulose can adsorb bilirubin molecules through electrostatic interactions, providing a research basis for using nanocellulose as a blood purification adsorbent. To further explore the potential of TOCN beads as a blood purification adsorbent, 10 mg of TOCN aerogel beads obtained after freeze-drying were first placed into a 10 mL syringe to simulate the blood perfusion process, as shown in Figure 10a. Bilirubin at a concentration of 0.03 mg mL^−1^ was dissolved in rabbit plasma, and then this plasma (10 mL) was added to the simulated blood perfusion device. Plasma without adsorbent was used as a blank control to calculate the bilirubin adsorption capacity and to study the dynamic adsorption process. Figure 10b shows the change in bilirubin concentration in rabbit plasma over time. After adsorption with TOCNB3, the bilirubin concentration in rabbit plasma decreased from 0.03 to 0.0089 mg mL^−1^ within 90 min, which is below the average bilirubin concentration in the human body (about 0.01 mg mL^−1^), representing a decrease of approximately 70%. The results demonstrate that TOCN beads have excellent blood compatibility, self-anticoagulation ability, and superior toxin removal capability, thereby providing a basis for the application of adsorbents in blood purification.

## 4. Conclusions

In this work, the structure and properties of TOCN beads with varying carboxyl contents were studied. The carboxyl content of TOCN was controlled by adjusting the amount of NaClO during the TEMPO oxidation process, and nanocellulose beads were prepared using the drop cure method. The results reveal that TOCN beads possess a uniform nanometer-scale three-dimensional porous structure, with a specific surface area and consistent pore size distribution as the carboxyl content rises, along with a uniform pore size distribution. FTIR and zeta potential analyses indicated that an increase in carboxyl content leads to higher carboxyl content and potential values. Adsorption experiments demonstrated that TOCN beads have a strong adsorption capacity for toxins such as bilirubin, creatinine, uric acid, and Cu^2+^, with maximum adsorption capacities of 310.6 mg/g, 16.3 mg/g, 127.51 mg/g, and 145.4 mg/g, respectively. TOCN beads also exhibit good blood compatibility, with a low hemolysis rate, and effectively restore bilirubin concentrations in blood, showing their potential as blood purification adsorbents. In the future, we will evaluate the toxin removal performance of TOCN beads in synthetic plasma supplemented with human serum albumin and in vitro human plasma to address protein-binding interference issues and further validate their potential for clinical application in blood.

## Figures and Tables

**Table 1:** Pseudo-first-order and pseudo-second-order kinetic model parameters of TOCN beads and bilirubin.

Sample		Pseudo-First-Order Model ln(q_e_ − q_t_) = lnq_e_ − k_1_t	Pseudo-Second-Order Model t/q_t_ = 1/k_2_q_e_^2^ + t/q_e_
	q_e_-exp (mg/g)	q_e_-cal (mg/g) k_1_ (min^−1^)	q_e_-cal (mg/g) k_2_ (min^−1^)
TOCNB1	265.85	179.19 0.0243	307.69 0.000136
TOCNB2	278.69	207.65 0.02351	319.4 0.000139
TOCNB3	288.91	175.74 0.02789	330.03 0.000157

**Table 2:** Parameters for Langmuir and Freundlich isotherms.

		Freundlich lnq_e_ = lnK_F_ + b_F_lnC_e_	Langmuir C_e_/q_e_ = C_e_/q_m_ + 1/q_m_K_L_
	q_e_-exp (mg/g)	k_F_ (mg g^−1^) b_F_	q_m_ (mg/g) k_L_ (L mg^−1^)
TOCNB1	265.85	1096.6 0.50707	285.7 0.068
TOCNB2	278.69	1002.2 0.45534	301.2 0.062
TOCNB3	288.91	796.3 0.37445	310.6 0.052

## Data Availability

The original contributions presented in this study are included in the article. Further inquiries can be directed to the corresponding authors.
